# Extreme environments offer an unprecedented opportunity to understand microbial eukaryotic ecology, evolution, and genome biology

**DOI:** 10.1038/s41467-023-40657-4

**Published:** 2023-08-16

**Authors:** Hannah B. Rappaport, Angela M. Oliverio

**Affiliations:** Department of Biology, Syracuse University, Syracuse, NY 13210 USA

**Keywords:** Microbial ecology, Microbial communities, Cellular microbiology, Genomics

## Abstract

Research in extreme environments has substantially expanded our understanding of the ecology and evolution of life on Earth, but a major group of organisms has been largely overlooked: microbial eukaryotes (i.e., protists). In this Perspective, we summarize data from over 80 studies of protists in extreme environments and identify focal lineages that are of significant interest for further study, including clades within Echinamoebida, Heterolobosea, Radiolaria, Haptophyta, Oomycota, and Cryptophyta. We argue that extreme environments are prime sampling targets to fill gaps in the eukaryotic tree of life and to increase our understanding of the ecology, metabolism, genome architecture, and evolution of eukaryotic life.

## Abstract (web summary)

The ecology and evolution of eukaryotic microbes in extreme environments are poorly understood. In this Perspective, Rappaport and Oliverio summarize data from over 80 studies of protists in extreme environments and identify lineages of particular interest as targets for future research.

## Introduction

The study of microbes that inhabit extreme environments has revealed a great number of phylogenetically novel and metabolically diverse microbial lineages^1^. That extreme environments have shed light on previously dark areas of the Tree of Life is not a coincidence: these ecosystems span dramatic ranges of geochemistry, including temperature, pH, and salinity^2,3^ and physical formations such as hydrothermal vents, geothermal springs, soda lakes, acid mine drains, solar salterns, and the cryosphere (glaciers and permafrost). Many such environments are also host to multiple extreme conditions. For example, geothermal springs can be in both high-temperature and low-pH environments. The unique geochemical conditions found in extreme environments host a similarly unique diversity of life. For instance, nearly all the candidate archaeal phyla have been recovered from extreme environments^4^.

Despite the importance of extreme environments to our understanding of microbiology, ecology, and evolution, protists have been largely ignored. Protists are single-celled eukaryotic organisms (excluding fungi) with diverse morphologies and functional strategies. Although they represent the vast majority of eukaryotic phylogenetic diversity^5,6^, protists remain underrecognized in extreme environments. This is true even relative to other eukaryotes such as fungi, despite mounting evidence that protists are active and widespread in extreme conditions.

**Fig. 1:**
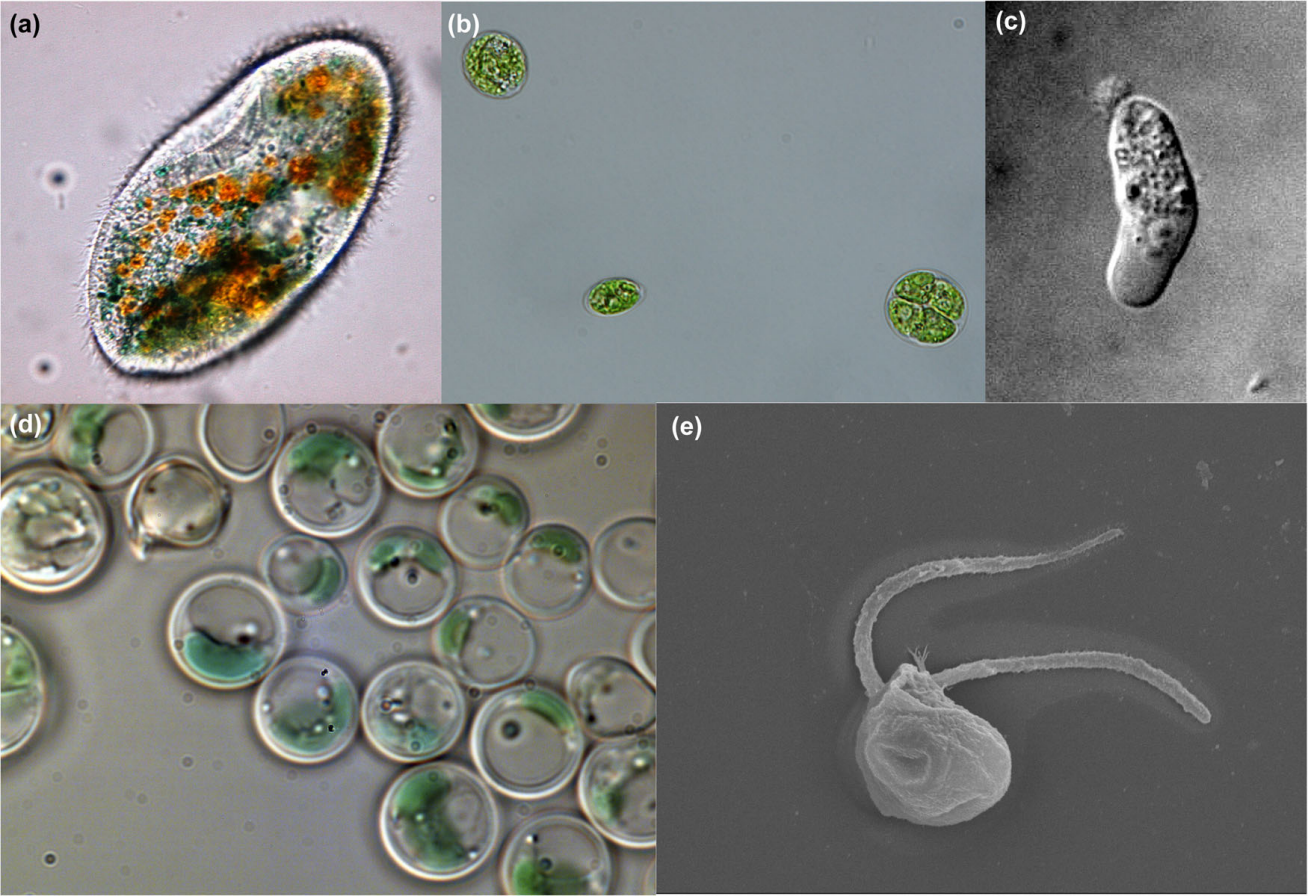
Examples of protists highlighting the morphological diversity of extremophiles. **a** *Frontonia*, an alkaliphilic ciliate from soda lakes in Kenya. Light microscope image (×400) courtesy of Geoffrey Odhiambo Ong’ondo (Egerton University). **b** *Chlamydomonas pitschmanii* Ettl, a strain of thermoacidophilic green algae isolated from hot spring soils. Light microscope image courtesy (×100) of Antonino Pollio (University of Naples Federico II). **c** *Tetramitus thermacidophilus* strain BSL, an amoeboflagellate from an acidic geothermal lake in California, USA. DIC image (×1000) courtesy of Gordon Wolfe (California State University, Chico), photographed by Billie Reeder. **d** *Galdieria sulphuraria*, a mixotrophic species of thermoacidophilic red algae, here growing under illumination. Light micrograph courtesy of Gerald Schöenknecht (Oklahoma State University, Heinrich-Heine-Universität Düsseldorf). **e** *Halocafeteria seosinensis*, a halophilic, heterotrophic nanoflagellate isolated from a saltern in Korea. SEM image (×10,000) courtesy of Jong Soo Park (Kyungpook National University).

Extremophile protists represent diverse morphological forms as well, including ciliates (Fig. 1a), algae (Fig. 1b, d), amoebae and amoeboflagellates (Fig. 1c), and flagellates (Fig. 1e), among others. In part, the neglect of extremophile protists is due to a longstanding assumption that microbial eukaryotes are poorly suited to life in extreme conditions. Additionally, many protists are notoriously challenging to culture^7^ and to study with genomic methods due to their often large and complex genomes^8^. In extreme habitats, technological challenges present additional difficulty: physical access to extreme environments is often complicated^9^, and study material may be limited. Furthermore, rapid changes in environmental conditions including pressure or temperature due to sampling may compromise eukaryotic cells^10^. However, current developments in sequencing technology and corresponding bioinformatics tools^11^ provide new opportunities to study microbial eukaryotes. Recent studies have started to reveal how phylogenetically diverse eukaryotes cope with physiological stresses such as extreme acidity and alkalinity, extremely hot and cold temperatures, and high salinity environments^12–14^.

To advance the study of microbial eukaryotic diversity at a time when an increasing number of protists are being discovered and studied via multi-omics and cultivation, we evaluate the distribution, ecology, diversity, and genomic traits of extremophile protist lineages. In the first section, we synthesize available culture data from over 50 representative extremophilic protist isolates towards a comprehensive understanding of the known growth optima and limits of protist life (for the full list of isolates, see Supplementary Dataset 1). We focus on the extremes of temperature, pH, and salinity here, while acknowledging that there are other extreme conditions protists inhabit. For example, there is substantial existing literature on anaerobic protists^15–20^, detailing the fascinating adaptations to life in low or no oxygen environments. Likewise, extreme conditions can co-vary; high pressure is associated with high-temperature hydrothermal vents, high concentrations of heavy metals are often a component of highly acidic environments, and extreme salinities and temperatures are associated with alkaline environments^21^.

In the second section, we complement the culture-based data by including sequencing data from over 80 studies that span geothermal springs, hydrothermal vents, soda lakes, acid mine drainage, solar salterns, and cryosphere environments to contextualize our understanding of protist diversity in extreme environments within a phylogenetic framework. For example, we include sequences from a recent survey from deep-sea hydrothermal vents in the Northeast Pacific Ocean^12^ and from a gradient of geothermal springs in New Zealand^13^ (for a complete list of environmental sequencing studies included, see Supplementary Table 1, and for sequence taxonomy, see Supplementary Dataset 2). From these data, we identify understudied groups of extremophilic protists, including many unknown lineages that are strongly associated with a particular extreme condition. In our third section, we highlight genome features of extremophilic protist lineages, including horizontal gene transfer (HGT) as well as genome reduction, genome evolution, and gene family expansion. Given recent methodological and conceptual advances, the field of extremophile biology is poised to rapidly broaden our understanding of microbial eukaryotic ecology and evolution. We conclude with promising future research directions, including using extreme environments as model systems to study ecological interactions, sampling novel and potentially early-diverging lineages to inform our understanding of the tree of life, and applying comparative genomics to uncover potential mechanisms of adaptation to these unique habitats.

## Protists can survive in a broad range of extreme conditions

**Fig. 2:**
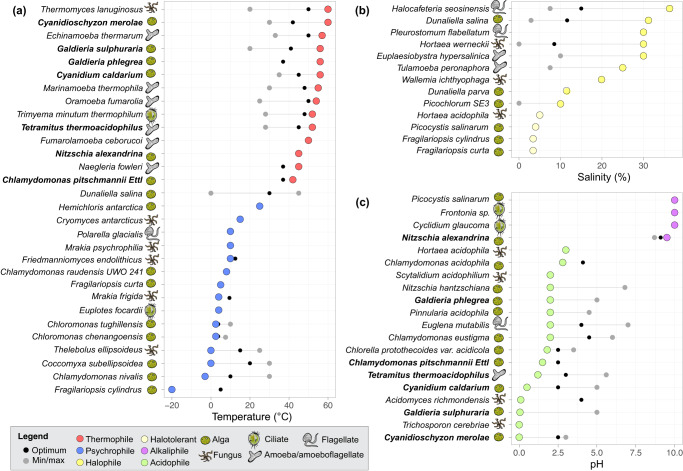
Extreme conditions tolerated by isolated protists. Dumbbell charts highlight the condition optima (average of optimal ranges when available, or sampling condition when culturing conditions were not available) and growth ranges of representative isolates by **a** temperature, **b** salinity, and **c** pH. For all, the extreme maximum/minimum condition values are colored by extremophile type. Isolates are also labeled by morphological form, including amoeba, alga, ciliate, flagellate, amoeboflagellate, and fungi for comparison based on direct references to the isolates’ form or otherwise the major form of the clade it falls in. Algae include diatoms, fungi include yeasts, and flagellate includes dinoflagellates. Isolate names in bold are polyextremophiles (*n* = 7), shown in multiple subplots (for a complete list of isolates, see Supplementary Dataset 1).

A growing list of protist lineages has been isolated from extreme environments^22–26^, enabling a more comprehensive understanding of the degree to which morphological and phylogenetically diverse protists can withstand a broad range of extreme conditions. These efforts have also started to establish how the growth optima and limits of protists in extreme conditions (including high and low temperature, high and low pH, and high salinity) compare to those of bacteria, archaea, and fungi. To highlight our current understanding of growth optima and limits across extremophilic protist lineages, we compiled ranges of temperature, pH, and salinity for 51 representative protist isolates from extreme environments (Fig. 2), including some of the most extremophilic fungal species to contextualize protists within the broader eukaryotic framework. Below, we summarize the trends in protist isolate growth ranges and optima across extreme environmental conditions including temperature, pH, and salinity (see Table 1 for range definitions for each condition).

**Table 1:** Glossary

Term	Definition
Acidophile	Organism with optimal growth at pH <5, associated with heavy metal conditions as well^52^. We define moderate acidophile as having optimal growth between pH 5 and 6.
Algae	Polyphyletic group of typically photosynthetic organisms with representatives across multiple divisions including Chlorophyta, Rhodophyta, and Ochrophyta. We focus on single-celled microalgae.
Alkaliphile	Organism with optimal growth at pH > 9^52^
Amoeba	Morphological form with amoeboid movement (changes shape and moves with pseudopods/“false feet”), found across multiple divisions including Lobosa (Amoebozoa) and Discoba (Excavata).
Amoeboflagellate	Morphological form that includes both an amoeboid and flagellate state
Ciliate	Clade of alveolates in the phylum Ciliophora that have hair-like organelles called cilia
Flagellate	Morphological form that moves with tail-like structures called flagella, found across multiple divisions including Opalozoa, Dinoflagellata, and Discoba
Fumarole	Vent near volcano, high-temperature conditions
Halophile	Organism adapted to high salinity. Extreme halophiles grow in 14.6–30.4%^96^. Moderate or borderline halophiles grow in 8.8–23.4% salinity. Halotolerant organisms grow in moderately saline conditions: 2.9–14.6% salinity^96^. For reference, the ocean has a salinity of 3.5%
Geothermal spring	Body of water heated geothermally, often associated with high temperatures and a range of pH levels. Also known as hot springs
Hydrothermal vent	An area of seafloor with an opening heated geothermally, associated with high temperatures
Multi-omics	The application of multiple ‘omics technologies (ex. genomics, transcriptomics, proteomics, metabolomics).
Polyextremophile	An organism that thrives in multiple extreme conditions (ex. High temperature and low pH)
Protist	Microbial (typically unicellular) eukaryotic organisms excluding animals, plants, and fungi.
Psychrophile	Organisms with optimal growth below 15 °C, maximum growth around 20 °C, and minimum around 0 °C or lower^97^. We counted organisms as psychrophiles that met at least one of these conditions and considered moderate psychrophiles as having optimum growth ≤20 °C. We also included cryophiles, organisms associated with snow, as psychrophiles.
Soda lake	Water body with alkaline conditions
Thermophile	Organisms with growth above 40 °C^98^. We considered moderate thermophiles as having growth between 37–40 °C.

A suite of thermophilic protist lineages can grow at temperatures above 50 °C. Algae, along with fungi, are among the most well-studied eukaryotic organisms in extreme environments. Collectively, they represent over 75% of the species for which we were able to obtain growth optima data. The red alga, *Cyanidioschyzon merolae* (Fig. 2), can persist up to 60 °C^27^ (the same maximum as the fungus, *Thermomyces lanuginosus*^28^). Other red algae, including *Cyanidium caldarium, Galdieria sulphuraria*, and *Galdieria phlegrea*, are also able to persist at maximum temperatures of 56 °C^29–31^. These algae have a variety of strategies that enable withstanding high temperatures, including the production of thermostable enzymes and protective extracellular polymeric substances, as well as high rates of primary production which can fuel the development of these specialized features^32^. Similar strategies, such as the production of thermostable enzymes, have also been observed in bacteria^32^.

Whereas fungi and algae are well-known to withstand high temperatures, there are also poorly-characterized amoeboid lineages spanning multiple eukaryotic supergroups including Amoebozoa and Discoba. These phylogenetically diverse amoeboid lineages can persist in extremely hot environments; known species include *Echinamoeba thermarum* within Amoebozoa and *Tetramitus thermacidophilus* (Fig. 1c), *Marinamoeba thermophila*, *Fumarolamoeba ceborucoi*, and *Oramoeba fumarolia* within Discoba^22,25,33–36^. Amoebae may be particularly well-suited to thermophily and have also been repeatedly recovered from artificially heated systems in addition to naturally warm environments^25^. Researching the evolution of amoebae across multiple distantly related lineages in high-temperature environments will shed light on to what extent the adaptations that enable survival in hot conditions are linked to this morphological form. The functional contributions of thermophilic amoebae to the broader ecology of extreme environments also remain unclear. Beyond their roles as consumers of archaea and bacteria^37^, thermophilic amoebae may also play key roles as hosts to bacterial and viral endosymbionts and in some cases, potential pathogens in high-temperature environments. Characterized as ‘Trojan horses’, amoebae can host intracellular bacterial symbionts that survive phagocytosis^38^. Likewise, the discovery of giant viruses in the amoeba, *Acanthamoeba polyphaga*^39^, led to a major paradigm shift in the field of virology given their size and complexity^40^. Giant virus-like particles were found in *Acanthamoeba* isolates from Boiling Springs Lake, hinting at intriguing virus-amoebae interactions in extreme environments as well^33^, although viral-amoebae interactions remain mostly uncharacterized in high-temperature habitats.

Algae and amoebae are not the only morphological forms of protists that have been isolated from extremely hot environments, but they seem particularly successful in these environments, given their representation in thermophilic conditions across multiple distantly related lineages. Another protist, the anaerobic ciliate *Trimyema minutum thermophilum*, is also found in hydrothermal vents up to 52 °C^23^. *Trimyema* has an impressive optimal growth temperature of 48 °C, which is higher than the red algal isolates and many of the amoeboflagellates.

Extreme heat may be one of the only conditions where eukaryotes cannot match bacteria and archaea. While Archaea are the only hyperthermophiles, growing above 80 °C^41^, in extremely cold environments (the cryosphere), protists are remarkably successful. *Fragilariopsis cylindrus*, a polar diatom, can grow at temperatures down to −20 °C^42^. Within fungi, *Cladosporium herbarum* can withstand temperatures of −8 °C^43^. In bacteria, *Planococcus halocryophilus* Or1 grows and divides at temperatures as low as −15 °C and it is metabolically active down to at least −25 °C^44^. Among archaea, *Deinococcus geothermalis* DSM 11300 can also survive at −25 °C^45^. Algae also appear to be well suited to psychrophilic environments. For example, *Chlamydomonas nivalis* is an alga that creates red blooms and photosynthesizes in the snow (growth down to −3 °C^46^). Other algae, including *Coccomyxa subellipsoidea* (min 0 °C^47^), *Chloromonas tughillensis* (min 2.5 °C, and optimally at 2.5 °C or 5 °C^48^), and *Hemichloris antarctica*^49^ have also been cultivated from psychrophilic environments. Algae are the dominant phototrophs in some extremely cold environments like sea ice, which is most influenced by diatoms^50^, and alpine snow, which is dominated by the green algae, *Chloromonas* and *Chlamydomonas*^51^. Due to their coloring^48^, algae are readily visible in snow. It is likely with additional sampling effort that other, less conspicuous protists that are also well adapted to cold environments will be discovered. Regardless, it is already evident that protists play important roles as phototrophs in extremely cold environments.

Like extreme cold, protists can withstand similar extremes in pH and salinity relative to bacteria, archaea, and fungi^52,53^, and can also persist in polyextreme environments. Notably, Cyanobacteria cannot survive below pH 4, enabling algae to be the dominant primary producers in many extremely acidic habitats^54^. The optimal growth of representative protist acidophiles (Fig. 2) ranges between pH 4.5 to pH 2, with a recorded minimum growth of as low as pH 0 by the red alga *Cyanidioschyzon merolae*^45^. The lowest minimum recorded pH for fungi (*Trichosporon cerebriae*^53^) and archaea (*Picrophilus oshimae* and *P. torridus*) is also 0 (0.06 for the archaea)^45^. The red algae are well-known for their tolerance to low pH, even in high temperatures. For example, *C. caldarium* is the dominant primary producer in its polyextreme habitat^31^. Red algae are not the only protists to succeed in acidic hot environments, though. A green alga, *Chlamydomonas pitschmanii* Ettl, is found in high-temperature, low-pH geothermal spring soils^55^ (Fig. 1b). Additionally, the amoeboflagellate, *T. thermacidophilus* (Discoba: Heterolobosea) is another polyextremophile that inhabits high-temperature and acidic geothermal springs and fumaroles^36^.

It is already clear that alkaliphilic protists can withstand high pH environments, but the data are much more limited relative to protists in low pH environments. Alkaliphilic protists have been sampled up to at least pH 10.48^14^, and it is likely that the pH limit may be even higher with increased sampling effort. In comparison, the most alkaliphilic known bacterium was found at pH 12.5^45^. Alkaliphilic protists sampled are primarily represented by ciliates, but a diversity of eukaryotic phytoplankton have been found as well: *Cyclidium glaucoma* was the most abundant ciliate recovered in alkaline-saline lakes of Kenya^14^, and members of the genus *Frontonia* have been recovered from soda lakes in Turkey^56^ and Kenya^57^ (Fig. 1a). The low number of alkaliphile isolates compared to acidophiles does not necessarily indicate a difference in prevalence between acidophiles and alkaliphiles but rather may reflect a difference in sampling effort between these conditions^52^.

Protists are also remarkably successful in extreme salinity. Using the salt-out strategy, protists such as *Halocafeteria seosinensis* and *Dunaliella salina* accumulate compatible solutes which are small organic compounds present at high concentrations in the cell to balance out their hypertonic surroundings^58^. In contrast, many hypersaline bacteria use a salt-in strategy, where large concentrations of K^+^ and Cl^−^ ions accumulate in the cell^58^. While seawater has ~3.5% salinity^59^, the flagellate *Pleurostomum flabellatum* has an optimal salinity of up to 30%^60^. Another flagellate, *H. seosinensis*, can tolerate up to 36.3%^24^ (Fig. 1e). This is higher than the maximum salinity reported for bacteria, which is 35%^45^, and similar to fungi (*Wallemia ichthyophaga* can grow up to 30%^61^).

**Fig. 3:**
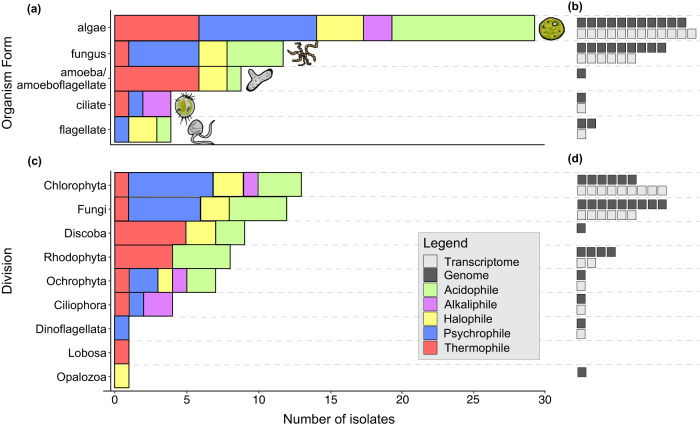
Algae have the greatest number of genome and transcriptome representatives within extremophile microbial eukaryotes. Bar charts summarize representative microbial eukaryotic isolates by **a** morphological form and **c** taxonomic division. Columns are colored by the extremophile type of the isolates represented. Algae include diatoms, fungi include yeasts, and flagellate includes dinoflagellates. Count of published genomes (from NCBI and JGI) for each morphological form **b** or division **d** is shown in dark gray boxes to the right of each column, and of transcriptome in light gray boxes. Polyextremophiles were counted for each condition (*n* = 7).

To further elucidate potential functional contributions, we also summarized available isolate data for different organism forms (Fig. 3a). For example, algae are known to be photosynthetic or mixotrophic, the fungi included are saprobes, and the flagellates, ciliates, and amoebae/amoboflagellates are associated with heterotrophy. From these data, we note a couple of emerging patterns. First, in high temperature, salinity, or pH environments, heterotrophs including amoebae, flagellates, and ciliates are often recovered. In low temperature and pH environments, algae are frequently found. That thermophily occurs in multiple, phylogenetically disparate amoeboid lineages and that psychrophily appears frequently across algal lineages may inform evolutionary trends and ecology in extreme environments. Protists have adapted to a remarkable range of conditions and may occupy even greater extremes; however, it is important to note that they remain severely under-sampled relative to other groups. Therefore, there are still critical gaps in our understanding of their physical limits and ecological roles in extreme environments.

Other ecological strategies, such as forming cysts and living within biofilms, may also aid in the persistence of protists when conditions are unfavorable. Many protists, including dinoflagellates^62^ and ciliates^63^ can withstand extreme conditions while inactive, using the strategy of encystment^64^. Dinoflagellate cysts are protective against nutrient, UV, and sometimes temperature stress^62^. These cysts have been described as microbial seed banks in environments like vernal pools, where conditions range from favorable to desiccated^65^. Another protective strategy is biofilm formation, exemplified by Cyanidiophyceae^66^. Biofilms are aggregations of microbes and extracellular polymeric substances and provide protection to the internal microbes from external conditions. *Dunaliella* and *Cyanidium* grow within biofilms in the extremely acidic Río Tinto^67^. Another striking example of protective biofilms in an extremely acidic environment is the slime streamers of the Königstein uranium mine, which host a diverse array of protists including flagellates, ciliates, and amoebae^68^.

## Phylogenetic analysis highlights extremophile protist diversity and identifies lineages for future research

**Fig. 4:**
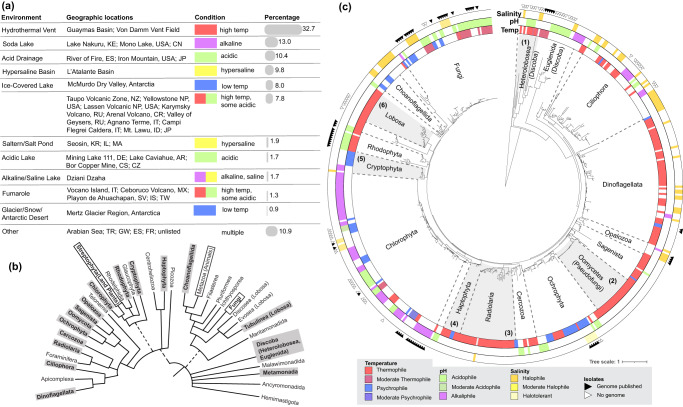
Phylogenetic tree of extremophilic sequences highlights novel and unknown lineages as promising candidates for further research. **a** The geographic locations and environments of the protist samples from temperature, pH, and salinity extremes are included in this review. The percentage column summarizes the sequences obtained for each extreme environment. **b** Cartoon tree of eukaryotes. Protist lineages with extremophile sequences sampled are highlighted in gray. **c** Phylogenetic tree built from 18 S rRNA includes environmental sequences from extreme environments (for a complete list of references, see Supplementary Table 1, and for taxonomy see Supplementary Dataset 2). We downloaded sequences from NCBI Genbank and Zenodo, aligned using Silva ACT with nearest neighbors, and trimmed using trimAl. A total of 530 sequences are included on the final tree built with RAxML-HPC BlackBox on CIPRES. We visualized the tree on iToL with neighbor sequences removed. Isolate sequences are marked with a black triangle if a published genome is available and a white triangle if no genome is available (from NCBI and JGI). Extreme conditions are annotated by rings for temperature (innermost), pH (middle), and salinity (outermost). The candidate extremophilic lineages identified are highlighted in gray with numbered brackets, and include: 1. Heterolobosea (Discoba), 2. Oomycota (Pseudofungi: Stramenopiles), 3. Radiolaria, 4. Haptophyta, 5. Cryptomonadales (Cryptophyta), and 6. Echinamoebida (Lobosa: Amoebozoa).

As most protists remain uncultured, environmental sequencing has been an important tool in revealing the diversity of protist lineages that can persist in extreme environments^12,14,69^. To provide an overview of broad patterns in protist phylogenetic diversity across extreme environments (Fig. 4a), we summarize available sequencing data (18 S rRNA gene sequences from both community samples and cultured isolates) from 81 studies in a phylogenetic tree (Fig. 4c). Data collection and tree-building details are included in Supplementary Methods. We relate the presence of lineages containing extremophiles to the broader eukaryotic tree of life and find that extremophiles are present widely across the tree (Fig. 4b). Most extremophilic lineages have been recovered only from environmental samples and do not have representative isolates, including lineages such as Cryptophyta, Haptophyta, and Radiolaria. In contrast, phylogenetic lineages with the greatest isolate and genome representation include Chlorophyta, Rhodophyta, and Opalozoa. Some lineages, such as Chlorophyta and Ochrophyta, are noted for their broad adaptation to multiple extreme conditions. There are also a multitude of lineages with high coverage in environmental studies from extreme conditions but lack isolates or published genomes (Fig. 4c, black triangles represent isolates with published genomes, and white triangles represent isolates without published genomes).

Based on the phylogenetic analysis, we identify lineages that are of significant interest for future research efforts. These are clades that have been detected across multiple studies, are strongly associated with a specific extreme condition, and for which there are currently no genomic or transcriptomic datasets. We identify six focal lineages associated with high temperature, including the Echinamoebida, Heterolobosea, Radiolaria, Haptophyta, and Oomyceta. Echinamoebida (Fig. 4c) are a clade of thermophilic amoebae within Lobosa (Amoebozoa), known to withstand up to 57 °C. Environmental sequences of *E. thermarum* have been recovered from distant geographic locations from geothermal springs of Taupō Volcanic Zone in New Zealand, Yellowstone National Park in the USA, Karymsky Volcano and Valley of Geysers in Russia, Arenal Volcano in Costa Rica, and Agnano Terme in Italy^13,25^. *E. thermarum-*like sequences have also been recovered from Black Canyon geothermal springs of the Colorado River, USA^70^. Environmental sequencing suggests that there may be multiple unique species within *Echinamoeba* as well as additional diversity at the genus and higher taxonomic levels^13^. Intriguingly, *Echinamoeba* has been detected in hospital hot water heating systems and may also be important components of the built microbiome^71^ (see Box 1 for an overview of extremophilic protists in the built microbiome). Surprisingly, there are no genomic or transcriptomic data for this lineage, despite its ubiquity.

There are numerous other amoeboid lineages that are worthy candidates for future research across a suite of extreme conditions (Fig. 4c). Within Discoba, there are several thermophilic species in the clade Heterolobosea that have been detected in multiple studies and from diverse geographic locations. For example, *T. thermacidophilus*, with an optimal temperature of 45 °C and pH 3, was isolated from fumaroles and hot springs^33,36^ and *Tetramitus-*like species were found in environmental samples of acid mine drainage^21,33,36^. Other understudied amoeboid lineages within Discoba include *M. thermophila*, *F. ceborucoi*, and *O. fumarolia*^22,34,35^. Although Heterolobosea includes the well-studied human pathogen *Naegleria fowleri*, most Heterolobosea remain unknown, with much to be uncovered about the lifestyles and adaptations of the highly thermophilic lineages within this clade. Heterolobosea also contains multiple halophilic lineages including *Euplaesiobystra hypersalinica* and *Tulamoeba peronaphora*, which are found at salinities up to 30% and 25% respectively^72^.

There are also several lineages within Radiolaria, Haptophyta, and Psuedofungi recovered from multiple hydrothermal vent systems that are only known via environmental sequencing. Sequences from Radiolaria (Rhizaria; Fig. 4c), planktonic organisms with beautifully intricate outer skeletons, are related to the Classes RAD-A, RAD-B, RAD-C, and Acantharea across at least three studies^12,73,74^. Radiolaria are well-studied in the fossil record because of their mineral skeletons but extant Radiolaria are understudied, partly because they have yet to be cultivated^75^. They are phagotrophs that often have symbiotic relationships with haptophytes or dinoflagellates^75^. Haptophyta (Fig. 4c) were some of the few lineages found across all vent sites in a broad survey of hydrothermal vent protists^12^. They are similarly known for their fascinating fossil record but like Radiolaria, extant species remain poorly described. Oomycetes (Pseudofungi; Fig. 4c) were also commonly found across hydrothermal vent sites in the same survey. While the clade of oomycetes is known to contain many parasites, including the causative agent of the Irish Potato Famine^76^, it is unclear whether the extremophile oomycetes take on a free-living lifestyle and whether adaptations to a parasitic lifestyle relate to adaptations to living in extreme environments^77^.

Multiple lineages within Cryptophyta have been recovered from studies focused on extreme cold and alkaline conditions (Fig. 4c), suggesting that these photosynthetic algae may be particularly well-suited to these environments along with the better-studied Chlorophyta (green algae) and Ochrophyta (primarily diatoms). Cryptophyta were the dominant organisms recovered from a permanently ice-covered lake in the McMurdo Dry Valley of Antarctica^69^ and were represented in Kenya’s Lake Nakuru soda lake^14^. Their mixotrophic lifestyles, similar to some of the red algae in hot, acidic environments, likely supplement photosynthesis in their low-nutrient, extreme environments where light is limited during polar winter^69^.

Our phylogenetic analysis highlights the substantial diversity of extremophile protists that are only known through environmental barcode sequencing. Most published genomes and transcriptomes of extremophile protists are from algal lineages, similar to the division of the representative isolates (Fig. 3b, d). As a point of comparison, of the representative fungal isolates, 69% have published genomes, and for the best-represented algae, only 42% have published genomes. In contrast, the amoebae and amoeboflagellates only have published genomes for 12.8% of representative isolates, and the only published genome is from a human pathogen, *Naegleria fowleri*. Despite evidence of their presence in extremely hot environments from multiple studies, amoebae are considerably lacking in published genomes and transcriptomes. Other protists, including ciliates and flagellates, also remain underrepresented in terms of the relative number of genomes and transcriptome representatives within these lineages. Notably, even where isolates are represented by genomic or transcriptomic data, this only represents a very small fraction of diversity within the extremophile lineages.

Box 1 Extremophilic protists in the built environment The built environment, which is composed of structures and systems constructed by humans, provides an exciting opportunity to study extremophile protists in environments that are more easily accessed than many natural extreme habitats such hydrothermal vents or acid mines. The built environment hosts many physical environments with extreme conditions that are by design inhospitable to most microbial life and which select for lineages that can withstand a specific set of conditions^99^. For example, dishwashers and washing machine bleach reservoirs are extreme niches within human homes, although a subset of bacteria and archaea can nevertheless withstand these conditions^100^. Protists have been sampled from extreme built environments including hot water heaters and wastewater systems. Notably, several genera of amoeba including *Echinamoeba* have been sampled from hospital hot water systems^71^, and members of the Cyanidiophyceae have grown in wastewater treatment facilities^30^. Sampling from the built environment may improve accessibility and uncover novel lineages shaped by the many unique selective pressures of these environments.

## Genomic features of extremophilic protists

**Fig. 5:**
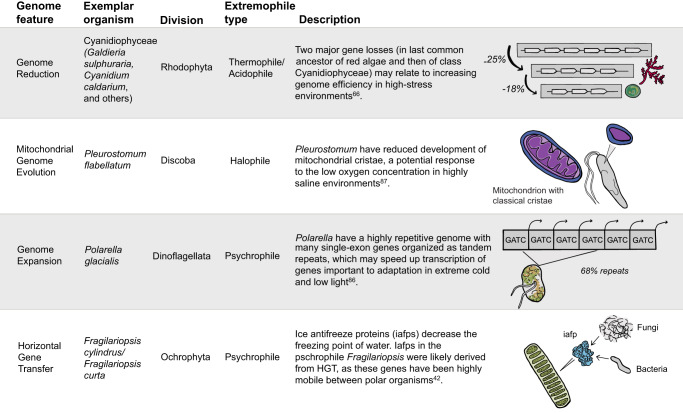
Genomic features of extremophile protists. We highlight notable genome attributes from extremophile protists across four main categories: genome reduction^66^, mitochondrial genome evolution^87^, genome expansion^86^, and horizontal gene transfer^42^. For a full summary of genome adaptations, see Supplementary Dataset 3.

The increasing application of microbial eukaryotic multi-omics approaches in the past few years is beginning to advance our understanding of the genomic attributes of microbial eukaryotic organisms in extreme environments^54,78,79^. Here, we summarize these into a toolkit of several key features detected across multiple lineages including (1) HGT, (2) genome expansion, (3) genome reduction, and (4) mitochondrial genome evolution (Fig. 5, Supplementary Dataset 3 for more information).

HGT has allowed many extremophile protists to benefit from the genetic adaptations and metabolic capabilities of extremophile bacteria, archaea, and other eukaryotes. This unique mechanism to accumulate genes laterally from other lineages rather than vertically may be more common in extremophiles, resulting from the pressures of introduction to new environments that close phylogenetic relatives may not be able to withstand^80^. While HGT has predominantly been recognized as a key genomic mechanism for adaptation within bacteria and archaea, extremophilic protists stand out from their examples of HGT in eukaryotes^80^. One of the foremost examples of HGT in microbial eukaryotes is the polyextremophilic Cyanidiophyceae. Horizontally transferred genes, mainly from bacteria and archaea, are thought to take up about 1% of the total genes on average in Cyanidiophyceae^66,81^. These include genes associated with the detoxification of heavy metals, the maintenance of proton pumps, and the ability to take up a greater variety of carbon sources^66,81^. Detoxifying heavy metals is critical to surviving low pH environments because acidic environments are associated with high concentrations of heavy metals like arsenic and mercury, and efficient proton pumps are essential by helping counter the pH stress of acidic conditions^66^. Likewise, the ability to uptake a variety of nutrient sources has enabled greater flexibility in resource utilization in stressful environments. For example, *G. phlegrea* live within rocks, which adds desiccation pressure, and have gained urease genes from bacteria via HGT, providing an alternate source of nitrogen^66,82^. Another member, *G. sulphuraria*, owes a remarkable 5% of its protein-coding genes to HGT^83^ (Fig. 1d).

There are many other examples of HGT in extremophile protists outside of the Cyanidiophyceae and in a variety of conditions. The acidophile, *Chlamydomonas eustigma*, acquired arsenic detoxification/biotransformation pathways via HGT, which likely aids in survival in low pH environments^84^. The halotolerant green alga, *Picochlorum* strain SE3, gained a variety of genes from HGT that are involved in metabolic flexibility and salinity tolerance^85^. In extremely cold environments, the diatom *F. cylindrus* acquired genes encoding antifreeze proteins, which decrease the freezing point of water, and the green alga^42^ *Chlamydomonas* sp. strain ICE-L gained similar ice-binding proteins^50^. These transferred genes can quickly provide beneficial adaptations that have previously evolved in other lineages alongside survival in extreme environments.

In addition to HGT, other genomic features may have helped protists to succeed in extreme environments. One example is gene family expansion, seen through genomic repeats and redundancy. This feature has been suggested to facilitate the evolution of adaptive gene functions that are not possible in smaller, functionally constrained genomes^84^. The genome of the dinoflagellate, *Polarella glacialis*, is made up of nearly 68% repeated regions. The genes are organized next to one another as tandem repeats, which may speed up transcription and increase efficiency in the cold, low light, high-stress environments they inhabit^86^. In *Chlamydomonas eustigma*, pathways involved in arsenic detoxification have been amplified, once again likely adding efficiency to the transcription of these essential genes for highly acidic environments^84^. *Euplotes focardii*, a psychrophilic ciliate, also has certain expanded gene families compared to its mesophilic relatives that are related to adaptation to its extreme conditions^78^.

Conversely, genome reduction, or gene loss, has also been detected in numerous extremophile protist lineages. A more streamlined genome could also increase efficiency in high-stress environments. The Cyanidiophyceae are a great example because while they have gained many genes through HGT, there have also been major gene losses in the evolution of the clade. About 25% of the genome was lost in the common ancestor of red algae and then another 18% was lost in the common ancestor of the Cyanidiophyceae^66^. A simpler body plan and lifestyle resulting from these losses are thought to be beneficial in the low nutrient, extreme environments these algae inhabit^66^. There were even further reductions in *G. phlegrea* and *Cyanidioschyzon merolae*, which lost over 1000 additional genes^82^. Outside of the Cyanidiophyceae, the green alga *Picochlorum* SE3, also has about 1500 fewer genes than its mesophilic sister taxon^85^. While genome reduction and expansion are traits within mesophilic lineages as well, future research could investigate whether these features are overrepresented among extremophiles. Reduction and evolution have not only occurred in the nuclear genomes of extremophile protists but also in organellar genomes of these lineages. For example, the halophilic flagellate, *Pleurostomum flabellatum*, lacks classical cristae in its mitochondria, potentially streamlining for limited aerobic use in the lower-oxygen, high-salt environments they inhabit^87^. In an observation from an extremophile’s mitochondrial genome, *G. sulphuraria* is known to have the highest mitogenome GC content of any known genome as well as a faster substitution rate than other red algae^88,89^. Critically, more protist genomes and transcriptomes are needed to determine if any of these observed genomic characteristics from a few studied extremophiles represent more generalizable features of eukaryotes in extreme environments and to assess broader phylogenetic and evolutionary trends in adaptation to extreme conditions. Likewise, beyond describing genomic attributes, documenting clear links between genotypes and phenotypes will be a critical step in describing and testing hypotheses about causal mechanisms of adaptation to extreme environments.

## Outlook

Although protists remain neglected in the study of life in extreme environments relative to archaea, bacteria, and even fungi, recent characterization efforts of extremophile protists have the potential to reshape our understanding of the ecological and molecular diversity of eukaryotic life. We suggest several exciting avenues to further this field of research.

Extreme environments hold great potential as natural model systems^1^, and here we suggest that they may be particularly well-suited to study the functional contributions of protists to ecosystems and their ecological interactions with bacteria, archaea, viruses, and fungi. There is generally lower biodiversity in extreme environments than in mesophilic environments due to ecological constraints^1,3,21^, meaning that ecological interactions can be studied at a scale with fewer variables. For example, a survey of Boiling Springs Lake in Lassen Volcanic National Park, USA, only found a single heterolobosean amoeba grazer in the most extreme conditions sampled, enabling a comprehensive description of the food web^33^. Metagenomics and metatranscriptomics approaches are particularly well-suited for extreme environments because of the low overall diversity of species within a site. Such approaches have already seen great success in other relatively low-diversity systems. For example, metatranscriptomics of marine protists led to the identification of genes associated with nutritional modes and ecological roles^90,91^. Likewise, extreme environments often span large ranges in environmental conditions within the range of a few meters^2,13^ and key parameters such as temperature and pH vary independently across features, enabling replicated sampling of dramatic environmental gradients that co-vary in most other natural systems. This makes extreme environments particularly good systems to disentangle the responses of specific organisms to abiotic conditions as well.

In addition, research on extremophilic protists can illuminate dark areas of the eukaryotic tree of life and provide insight into the early evolution of eukaryotes. Early-diverging protist lineages are likely to be overrepresented in extreme environments^92^. Inclusion of these early-diverging lineage is critical to phylogenetic reconstructions of the eukaryotic tree of life and could aid in the inference of characteristics of the last eukaryotic common ancestor^93^. Specifically, ancestral state reconstructions are highly sensitive to deep-branching lineages^92^. Additionally, these deep-branching lineages may help determine if certain eukaryotic genes or functions are archaeal in origin^94^. Excitingly, we have already started to see glimpses of phylogenetic discovery in recent studies. Many sequences from hydrothermal vents without close matches in existing databases are likely from early-diverging or novel lineages^12,95^. The Cyanidiophyceae, a thermoacidophilic class of red algae discussed above, is an early-diverging algal lineage proposed as a model group to study adaptations to early life on Earth, given their success in the similarly harsh conditions^66^. Research on Cyanidiophyceae (red algae) has highlighted the ability of protists to succeed in extreme conditions (particularly high temperature and acidity) and started to reveal the importance that HGT can have in the adaptation and evolution of eukaryotic organisms.

Applying similar efforts to a broad range of microbial eukaryotes will further broaden our understanding of the selective pressures that shape protistan life in these extreme environments. One approach that holds great promise is the use of comparative genomics to study extremophilic lineages in the context of closely related mesophilic species^66,78^. For example, the thermophile, *E. thermarum*, is closely related to a mesophilic species, *Echinamoeba exundans*^25^, and applying similar genomic approaches to what has been conducted in red algae could illuminate adaptations and underlying genomic signatures within this intriguing group.

Notably, our analysis suggests that amoeboid lineages, like red algae, may be particularly well-suited to life in high-temperature environments. Yet, amoeboid species remain among the least understood lineages within microbial eukaryotes, despite their tremendous morphological, phylogenetic, and metabolic diversity. Given that multiple lineages of amoebae have been repeatedly recovered from high-temperature environments, and that some lineages are already in culture, we argue that amoeboid lineages are obvious targets for genome sequencing and multi-omics profiling.

In summary, with this article, we aim to inspire increased sampling, isolation, cultivation, and multi-omics research on protists in extreme environments, guided by phylogenetic analyses to target major gaps in our current knowledge of extremophile diversity. This will yield the discovery of novel lineages, inform uncertain evolutionary relationships, and increase our understanding of the ecology, metabolism, physiology, and genome evolution of life on Earth.

### Supplementary information

Supplementary information

Description of Additional Supplementary Files - NEW

Supplementary Dataset 1

Supplementary Dataset 2

Supplementary Dataset 3

## Data Availability

The data included in this perspective are available within the supplementary files as well as on Figshare 10.6084/m9.figshare.22720309. NCBI accession numbers are listed where applicable.
