# Supplementary material for: Extreme environments offer an unprecedented opportunity to understand microbial eukaryotic ecology, evolution, and genome biology
Source: Nat Commun. 2023 Aug 16;14:4959. doi: 10.1038/s41467-023-40657-4 (PMC10432404; doi:10.1038/s41467-023-40657-4)
Supplement: Supplementary file 1 — Supplementary information [file 41467_2023_40657_MOESM1_ESM.pdf]

Extreme environments offer an unprecedented opportunity to understand microbial eukaryotic ecology, evolution, and genome biology

Hannah B. Rappaport[1] and Angela M. Oliverio[1]

1. Department of Biology, Syracuse University, Syracuse NY 13210, USA

This file contains:
- Supplementary Methods
- Supplementary References
- Supplementary Table 1

Supplementary Methods

All data and corresponding references obtained as a part of the methods described below have been deposited to Figshare, https://figshare.com/s/af555a0b67e2f1c0e91d. First, to highlight where data exist and are lacking for microbial eukaryotic presence in a suite of diverse extreme conditions, we first synthesized studies that reported protists' abilities to withstand extreme conditions (including high temperature, low temperature, high pH, low pH, and high salinity). For all isolates (n = 51), we included the condition optimum, minimum, and/or maximum where reported, along with the study reference (see Supplementary Table 1). These data were compiled to generate Figures 2 and 3. In Figure 3, we summarized the isolates by organism form and division. Form included amoeba, alga, ciliate, flagellate, amoeboflagellate, or fungus, and was based on direct references to form in the literature or by the major form of the clade in which the organism belongs. Division was listed by pr2 protist database taxonomic output[1]. For full taxonomic output, see Supplementary Dataset 3.

To estimate the number of transcriptomes and genomes that have been generated for extremophile protists (Figure 3), we searched for each isolate in both NCBI Taxonomy Browser[2] as well as JGI Gold (Genomes Online Database)[3]. We tallied both complete projects and permanent drafts. For isolates with published genomes, we conducted a literature review for published genome adaptations associated with extreme environments (see Supplementary Dataset 4).

To highlight the phylogenetic breadth of protists recovered from extreme environments, we built a tree of 18S rRNA gene amplicon sequences, as most protists (and particularly those in extreme environments) are only known through environmental sequencing. Importantly, our phylogenetic tree is meant to highlight representative protist diversity across extreme environments, rather than to present an accurate reconstruction of phylogenetic relationships. The protistan sequencing data presented here include both environmental sequencing of extreme environments[4,5] and 18S gene sequences from cultured isolates where possible. We included at least one representative community study from each environmental condition (including high temperature, low temperature, high pH, low pH, and high salinity). Studies were chosen based on having published raw 18S sequencing data from an extreme condition, where published raw data was a limiting factor. We also modeled a cartoon tree of eukaryotes off a cartoon tree in Jamy *et al.* 2022[6] to give more perspective to the diversity of extremophile protists and to the abundance of protist clades in comparison to plants, animals, and fungi. Isolate sequences for each extreme condition were identified via literature searches and are exemplar species of protists in each extreme condition. For all sequences included, please see Supplementary Dataset 2 for environmental sequences and Supplementary Table 1 for isolate sequences.

Sequences were downloaded from NCBI GenBank and Zenodo and classified using DADA2 with the pr2 protist database in R studio[1,7]. Any metazoan sequences were removed from community studies, as well as any sequences sampled from moderate conditions, wherever labeled from broader sampling efforts. We also subsampled our hydrothermal vent sequences as to not overrepresent one study[5] which had much more data than the others included. We took 9% of sequences from each taxonomic division sampled (Von Damm X18 site) and one sequence from each division if there were fewer than ten sequences overall to target 100 sequences overall from the study. The resulting sequences were compiled, and the nearest

neighbor for each sequence was found using Silva Incremental Aligner (SINA v1.2.12) "search and classify" default parameters to build a phylogeny with more confidence. We aligned the nearest neighbors with the original sequences using SINA with default parameters[8]. Gaps in the alignment were trimmed with trimAl (threshold = 0.2)[9]. We built a tree on the CIPRES Science Gateway (https://www.phylo.org/), using RAxML-HPC BlackBox with default parameters for nucleotide trees[10, 11]. We visualized the tree with iTOL (Interactive Tree of Life), where we removed nearest neighbor sequences and outliers (out of place based on DADA2 taxonomy) and then annotated tips based on isolate status, extreme condition, and genome presence[12].

Supplementary References

1. Guillou, L *et al.* The Protist Ribosomal Reference database (PR2): a catalog of unicellular eukaryote Small Sub-Unit rRNA sequences with curated taxonomy. *Nucleic Acids Res*. **41**, D597–604 (2013).
2. Schoch C*L, et al.* NCBI Taxonomy: a comprehensive update on curation, resources and tools. *Database* **2020** (2020).
3. Mukherjee, S. *et al.* Twenty-five years of Genomes OnLine Database (GOLD): data updates and new features. *Nucl. Acids Res* **51**, D957–D963 (2022*).*
4. Baker, B. J. *et al.* Metabolically Active Eukaryotic Communities in Extremely Acidic Mine Drainage. *Applied and Environmental Microbiology* **70**(10), 6264–6271 (2004).
5. Hu, S. K. *et al.* Globally-distributed microbial eukaryotes exhibit endemism at deep-sea hydrothermal vents. *Molecular Ecology* (2022).
6. Jamy, M. *et al.* Global patterns and rates of habitat transitions across the eukaryotic tree of life. *Nat Ecol Evol* **6**, 1458–1470 (2022).
7. Callahan, B. J., *et al.* DADA2: High-resolution sample inference from Illumina amplicon data. *Nature Methods* **13**, 581-583 (2016).
8. Pruesse, E. *et al.* SINA: Accurate high-throughput multiple sequence alignment of ribosomal RNA genes. *Bioinformatics* **28**, 1823–1829 (2012).
9. Capella-Gutiérrez, S. *et al.* trimAl: A tool for automated alignment trimming in large-scale phylogenetic analyses. *Bioinformatics* **25**, 1972–1973 (2009).
10. Miller, M.A. *et al.* Creating the CIPRES Science Gateway for inference of large phylogenetic trees. Proceedings of the Gateway Computing Environments Workshop (GCE), 14 Nov. 2010, New Orleans, LA, 1 - 8 (2010).
11. Stamatakis, A. RAxML Version 8: A tool for Phylogenetic Analysis and Post-Analysis of Large Phylogenies. *Bioinformatics* **30**, 1312–1313 (2014).
12. Letunic, I and Bork, P. Interactive Tree Of Life (iTOL): an online tool for phylogenetic tree display and annotation*. Bioinformatics* **23,**127-8 (2006).

Supplementary Table 1 – Environmental Sequences

Sources of environmental 18s rRNA sequences included in the review, with number of sequences included, environment, sampling condition, and geographic locations.

Number of Seqs. Included	Environment	Condition	Condition specifics	Geographic location	Reference
100	hydrothermal vent	high temp	48°C	MCR Von Damm HOG X18 site	Hu, S. K., Smith, A. R., Anderson, R. E., Sylva, S. P., Setzer, M., Steadmon, M., Frank, K. L., Chan, E. W., Lim, D. S. S., German, C. R., Breier, J. A., Lang, S. Q., Butterfield, D. A., Fortunato, C. S., Seewald, J. S., & Huber, J. A. (2022). Globally-distributed microbial eukaryotes exhibit endemism at deep-sea hydrothermal vents. Molecular Ecology, mec.16745. https://doi.org/10.1111/mec.16745
74	hydrothermal vent	high temp	up to 45°C	Guaymas Basin	Edgcomb, Virginia P., et al. "Benthic Eukaryotic Diversity in the Guaymas Basin Hydrothermal Vent Environment." Proceedings of the National Academy of Sciences, vol. 99, no. 11, May 2002, pp. 7658–62, https://doi.org/10.1073/pnas.062186399.
63	soda lake	alkaline	pH 10.48	Lake Nakuru, KE	Luo, W., Kotut, K., & Krienitz, L. (2013). Hidden diversity of eukaryotic plankton in the soda lake Nakuru, Kenya, during a phase of low salinity revealed by a SSU rRNA gene clone library. Hydrobiologia, 702(1), 95–103. https://doi.org/10.1007/s10750-012-1310-y
53	hypersaline basin	hypersaline	36.5% salinity	L'Atalante basin	Alexander, E., Stock, A., Breiner, H.-W., Behnke, A., Bunge, J., Yakimov, M. M., & Stoeck, T. (2009). Microbial eukaryotes in the hypersaline anoxic L'Atalante deep-sea basin. Environmental Microbiology, 11(2), 360–381. https://doi.org/10.1111/j.1462-2920.2008.01777.x
42	ice-covered lake	low temp	max: 5°C	McMurdo Dry Valley, Antarctica	Bielewicz, S., Bell, E., Kong, W., Friedberg, I., Priscu, J. C., & Morgan-Kiss, R. M. (2011). Protist diversity in a permanently ice-covered Antarctic Lake during the polar night transition. The ISME Journal, 5(9), 1559–1564. https://doi.org/10.1038/ismej.2011.23
31	acid drainage	acidic	pH 2	River of Fire, ES	Amaral Zettler, L. A., Gómez, F., Zettler, E., Keenan, B. G., Amils, R., & Sogin, M. L. (2002). Eukaryotic diversity in Spain's River of Fire. Nature, 417(6885), 137–137. https://doi.org/10.1038/417137a
22	acid drainage	acidic, moderate temp	37°C, pH 1.4	Iron Mountain, USA	Baker, B. J., Lutz, M. A., Dawson, S. C., Bond, P. L., & Banfield, J. F. (2004). Metabolically Active Eukaryotic Communities in Extremely Acidic Mine Drainage. Applied and Environmental Microbiology, 70(10), 6264–6271. https://doi.org/10.1128/AEM.70.10.6264-6271.2004

20	geothermal spring	acidic, high temp	Springs sampled in environmental gradients up to 64.9°C, down to pH 2.02	Taupo Volcanic Zone, NZ	Oliverio, A. M., Power, J. F., Washburne, A., Cary, S. C., Stott, M. B., & Fierer, N. (2018). The ecology and diversity of microbial eukaryotes in geothermal springs. The ISME Journal, 12(8), 1918–1928. https://doi.org/10.1038/s41396018-0104-2
