# Supplementary material for: Extreme environments offer an unprecedented opportunity to understand microbial eukaryotic ecology, evolution, and genome biology
Source: Nat Commun. 2023 Aug 16;14:4959. doi: 10.1038/s41467-023-40657-4 (PMC10432404; doi:10.1038/s41467-023-40657-4)
Supplement: Supplementary file 2 — Description of Additional Supplementary Files - NEW [file 41467_2023_40657_MOESM2_ESM.pdf]

Description of Additional Supplementary Files:

Supplementary Data 1: References for the cultivation conditions for each representative isolate are listed, including sampling location, GenBank Accessions, number of sequences included, and presence of published genome, if available.

Supplementary Data 2: Taxonomic output and 18S sequence from DADA2 PR2 database for each sequence included in phylogenetic tree.

Supplementary Data 3: References of known genome adaptations of extremophile protists, with their form, taxonomic division, and extremophile type.
